# Corrosion Performance and Mechanical Strength in Aluminum 6061 Joints by Pulsed Gas Metal Arc Welding

**DOI:** 10.3390/ma15186226

**Published:** 2022-09-08

**Authors:** Isidro Guzmán, Everardo Granda, Celso Cruz, Dora Martínez, Benjamín Vargas, Jorge Acevedo, Gilberto Cruz, Yuliana Avila, Ruben Velazquez, Leonardo Flores

**Affiliations:** 1 Facultad de Sistemas, Universidad Autonoma de Coahuila, Unidad Arteaga, Saltillo 25350, Mexico; 2 Centro Universitario UAEM Atlacomulco, Universidad Autónoma del Estado de Mexico, Toluca 50000, Mexico; 3 Dirección de Manufactura, Centro de Ingeniería y Desarrollo Industrial, Querétaro 76125, Mexico; 4 Facultad de Ingeniería Mecánica y Eléctrica, Universidad Autónoma de Nuevo León, San Nicolás de los Garza 66451, Mexico; 5 Departamento Metal Mecánica, Instituto Tecnológico de Tlalnepantla, Tecnológico Nacional de Mexico, Tlalnepantla de Baz 54070, Mexico; 6 Centro de Investigación en Geociencias Aplicadas, Universidad Autonoma de Coahuila, Unidad Norte, Nueva Rosita 26830, Mexico; 7 Estudios y Análisis de Materiales S.A. de C.V., Monterrey 64010, Mexico

**Keywords:** aluminum 6061, weld joint design, GMAW-P, mechanical strength, corrosion performance, heat input, metallography, materials characterization

## Abstract

In this paper, the analysis of electrochemical corrosion performance and mechanical strength of weld joints of aluminum 6061 in two-heat treatment conditions was performed. The joints were produced by gas metal arc welding in pulsed mode. The original material exhibited precipitates of β and β” phases in a volume fraction (Vf) of 2.35%. When it was subjected to a solubilization process, these phases were present in a Vf = 2.97%. This increase is due to their change in shape and distribution in clusters within the aluminum matrix. After the welding process, the best sample in the solubilization condition reached 117 MPa, while the original material achieved 104 MPa, but all samples showed a fracture in the fusion zone. This is attributed to the heat input that produces high and low hardness zones along the heat-affected zone and the welding zone, respectively. Moreover, the change in microstructure and phase composition creates a galvanic couple, susceptible to electrochemical corrosion, which is more evident in the heat-affected zone than in the other weld regions, exhibiting uniform and localized corrosion, as was evident by electrochemical impedance spectroscopy. The heat from the welding process negatively affects the corrosion resistance, mainly in the heat-affected zone.

## 1. Introduction

The AA6000 series alloys are precipitation hardening aluminum alloys. They are generally known for their lightweight, high corrosion resistance, and good weldability [1,2]. Among the common uses of the AA6061 alloys, the automotive industry explodes its use due to its lightweight, which translates into reducing CO_2_ emissions to the atmosphere thanks to the fuel savings; also, it is used in the aerospace industry due to its excellent thermal and electrical conductivity properties, being lightweight, and its corrosion resistance [1,3,4].

Precipitation hardening alloys have dispersed particles or phases (dispersoids) within the aluminum matrix, which affect the mechanical resistance of the material (positively or negatively). Alloys of type AA6061 are characterized by the presence of β precipitation hardening phase, which contains elements, including Al, Si, and Mg, which promote the mechanical resistance of the material [5].

In the matrix microstructure, dispersoids are present in both coherent and incoherent phases. Hakem et al. [6] studied the precipitation mechanism of the β phase in an AA6061 alloy during a welding process. They established that the hardening beta phase is formed in the following sequence: α- Super Saturated Solid Solution → Clusters of Mg and Si (Guinier–Preston zones, GP) → metastable β” (coherent) → metastable β’ (semi-coherent) →β stable phase (Mg_2_Si, incoherent). This transformation sequence has been demonstrated by Yang et al. [7] using Transmission Electron Microscopy. The maximum amount of precipitates within the matrix is related to the highest hardness of the alloy [8,9,10,11] and the movement of dislocations during precipitate formation [12,13,14].

Safyari et al. [15] in their investigation on the influence of coherent and incoherent dispersoids in the hydrogen-induced embrittlement of high-strength aluminum alloys, found that coherent dispersoids (Al_3_Zr) increase the embrittlement resistance of the alloy in comparison with the incoherent dispersoids (Al_18_Mg_2_Cr_3_). In addition to affecting the resistance to hydrogen embrittlement, these particles affect the mechanical properties since the results showed that the ductility of the material with incoherent dispersoids is lower than that of the material with coherent particles. They explained this effect due to the formation of Orowan dislocation loops around the incoherent particles, which are bypassed by moving dislocations. In contrast, the coherent dispersoids have minimal contribution to the dislocation process.

The presence of coarse particles also deteriorates the mechanical resistance of the material. Contrarily to fine particles, coarse particles are insoluble at high temperatures and induce decohesion in the matrix. It causes low hardness due to partial dissolution, over-aging, and uncontrolled reprecipitation of the hardening precipitates during the thermal cycle [16].

Therefore, during welding, the heat coming from the process could produce both incoherent dispersoids and coarse particles, which can detriment the mechanical properties of the weld joint. Specifically, in the 6000 series aluminum alloys, Al-Fe-Si intermetallics produce multiple stoichiometric phases acting as nucleation particles to form Mg/Si precipitates having an essential role in the mechanical resistance of the alloy [17].

The gas metal arc welding (GMAW) process in pulsed metal transfer mode (also identified as GMAW-P) has been used for joining parts of AA6061 alloy because it shows excellent weldability and favors better mechanical properties [18]. The latter has been demonstrated in recent investigations, such as that of Patel et al. [19]. They studied the effect of the current and the angle of the torch in the application of welding in an AA6061–T6 alloy in plates with a thickness of 6.35 mm. When they applied a welding current in the range from 100 to 120 A at a constant voltage of 23 V and 90° in the angle of inclination of the torch, the best penetration is obtained by using a 4043–ER electrode as filler metal. Recrystallization close to the fusion zone occurred while elongated grains in the base metal appeared due to the heat input from the process.

Similarly, Chikhale et al. [20] studied the micro-structural characteristics when applying the GMAW–P process and a 4043–ER electrode, identifying different regions, such as Heat-Affected Zone (HAZ), Welding Zone (WZ), and Base Metal (BM). They found a tensile strength of 176 MPa and an average hardness ranging from 62 to 70 HV (Hardness Vickers) in samples at a welding current of 175 A.

Although the welding processes had obtained good mechanical properties, they usually cause a detriment in the corrosion resistance of the materials. Mathivanan et al. [21] observed the dissolution of precipitates in the HAZ, as reported by other authors also [6,22], causing a decrease in hardness and mechanical resistance in this area. Once a Tungsten Inert Gas (TIG) soldering process was performed, they found that the tensile strength was 196 MPa on average for all samples. Moreover, a corrosion test with an electrolyte with a pH of 5.9 composed of 58.5 g of NaCl and 10 mL of H_2_O_2_ in 1 L of H_2_O revealed that the area with the highest corrosion current density (Icorr); i.e., the lowest corrosion performance, was the welding zone (also known as the melting zone), exhibiting an Icorr value of 16 μA/cm^2^. The authors attribute this behavior to the tension and compression stresses in that zone.

Fahimpour et al. [23] compared the results obtained when joining an aluminum alloy through the friction welding process versus the Gas Tungsten Arc Welding (GTAW, also known as the TIG process) using a filler metal 4043–ER and aluminum base metal AA6061–T6. The corrosion test was performed with an aqueous solution of 3.5 wt.% of NaCl. They determined the corrosion rate in the welding zone and the base metal and deduced that the Icorr is higher in the weld due to the presence of Si-based eutectics and Fe-based precipitates formed during the fusion process. This shows that thermodynamically these structures are more susceptible to corrosion, and Fe precipitates act as cathodes in the corrosion process [24,25].

In this research, a semi-automatic GMAW welding process in pulsed metal transfer mode was performed on an AA6061–T6 aluminum in the as-received condition and with the alloy subjected to a solubilization heat treatment. The aim is to determine the corrosion performance in the heat-affected zone, base metal, and welding zone, as well as the tensile mechanical properties at the weld joint.

## 2. Materials and Methods

This section presents the details of the experiments and the characterization techniques. The materials used and their preparation, information on the welding process, the mechanical tests carried out on the joints, and the corrosion tests are explained.

### 2.1. Materials and Their Preparation

AA6061–T6 as-received aluminum plates were used as the base metal for experimentation. For the welding process, a filler metal 4043–ER was used. The chemical composition of the base metal and the filler material was measured by spark spectroscopy and is reported in Table 1.

In total, 16 samples of 150 mm in length, 100 mm in width, and 6.4 mm in thickness were cut from the AA6061–T6 base metal. They were V-grooved with a 45° bevel on one edge, so each plate joined to another beveled plate formed a welding coupon. A total of eight welding pairs were prepared. Eight plates were taken to form four welding coupons and were subjected to a T4 thermal treatment. It consists of a pre-treatment of solubilization and natural aging; the solubilization was carried out at a standard temperature of 530 °C with a soaking time of 3 h followed by cooling in water. Then, 4 samples were welded under the original T6 treatment and four more in the modified T4 thermal treatment.

### 2.2. Welding Process

After the preparation of the samples, the welding process was carried out following the combination of parameters enlisted in Table 2. The parameters chosen in this experiment were proposed in accordance with the literature and observations from preliminary results of stress and corrosion. Table 2 shows the sample identification (ID), the type of heat treatment (HT) applied to the plate, the welding current (*I*), the polarization voltage (*V*), and the welding application speed (WS). In addition, the input heat (Qnet) is exhibited, which was calculated by using Equation (1) [26].

(1) Qnet=ηQarc

being η the heat transfer efficiency (0.86 for GMAW [27]), and the arc energy Qarc is defined in Equation (2) [28]:

(2) $$Qarc=\frac{V\times I}{WS}$$

### 2.3. Mechanical Testing and Microstructural Study

**Figure 1:**
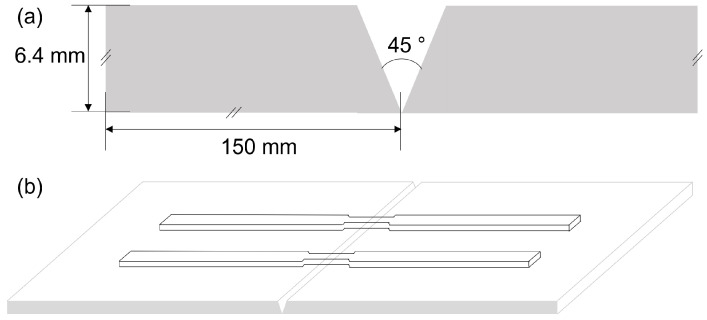
Detail of the V-grooved edges of the sample plates (**a**), and geometry and position of the tensile samples (**b**).

Once the GMAW-P process was finished, the welding samples were cut crosswise to obtain two specimens from each welded pair for the tensile testing (the AWS D1.2/D1.2M standard [29] indicates that the test must be evaluated as the average of two specimens: one sample and one replica). The tensile samples were machined according to the ASTM E8 standard [30] using the sub-size specimen specification, where the center of the specimen (gage length = 25 mm) is located in the center of the V-grooved edges of the joint (Figure 1a). The dimensions of the specimens were: overall length 100 mm, radius fillet 6 mm, reduced Section 32 mm, and width 6 mm. The tensile tests were carried out in an Instron 5980 tensometer. The weld was applied in the laminate direction of the aluminum plate.

The metallographic preparation of the samples was carried out based on the ASTM E3 standard [31]. It consisted of making a cross-section of the welded joint to obtain pieces that were mounted on Bakelite. These samples were roughened with abrasive SiC grinding paper in the following sequence of grit size numbers: 250, 600, 800, and 1200. Later, they were polished with a 1 μm diamond paste and 0.3 μm silica (SiO_2_) to obtain a mirror-like finish. Subsequently, the pieces were chemically etched by using hydrofluoric acid, according to ASTM E407 standard [32], to reveal the constituent microstructural phases of the base metal, filler material, and the different welding zones.

The observation by optical microscopy of the samples was performed using a Nikon Eclipse MA200 inverted-stage microscope and the NIS Elements D Nikon DS-03 software for capturing images of the different microstructural zones. The size of the metallic inclusions was measured in the two different heat treatment conditions, and the percentage of phases present in the weld was estimated using a Nikon stereoscope with the same image capture software. Additionally, a scanning electron microscope (SEM) Tescan Mira2 X-Sense was used to evaluate the surface of the sample, morphology, and distribution of metallic inclusions. Additionally, an energy dispersive spectroscopy (EDS) detector was used to determine the presence of constituent elements in the intermetallic particles.

In addition, to measure the microhardness in the different microstructural zones of the welded joint, a Vickers hardness test was carried out on the samples applying a 0.3 kgF load, using an Instron Tukon 2500 tester. Forty indentations were made along each piece per the ASTM E92 standard [33].

Finally, for the identification of phases, an Empyrean Panalytical X-ray Diffractometer (XRD) was used. The analysis was performed by X-ray Diffraction in microdiffraction mode on an area of 1 mm × 1 mm; the phase analysis was made by X’Pert HighScore Plus software and PDF+ XRD database, based on the charts from the ICDD (International Centre for Diffraction Data). A step size of 0.0016° with a counting time of 87.92 s, and CuK_α_ radiation (45 kV, 40 mA), were used. The analysis was performed in a 2θ range from 35° to 42° using the Rietveld refinement method [34,35].

### 2.4. Corrosion Tests

The selection of samples for the corrosion tests was based on the tensile test results: the best and worst conditions, i.e., the samples with the maximum and minimum tensile strength, respectively. Four samples were tested: the best and worst conditions from the samples joined with base metal AA6061–T6 and the best and worst conditions with base metal AA6061–T4.

Critical sections of the weld joint were cut using a diamond disc (fine cut), identified as Base Metal (BM), Heat-Affected Zone (HAZ), and Welding Zone (WZ). Once cut, electrical contact was made utilizing a copper cable welded to one of the faces of the sample using tin-lead welding, verifying that there was an electrical contact using a multimeter. Subsequently, each piece was cold-mounted to carry out the metallographic preparation of the area to be analyzed by roughing with the following SiC sandpaper sequence: 120, 240, 320, 420, 600, 800, and 1200. Finally, the surface was prepared to a mirror-like finish by polishing with cloth and alumina with a particle size of 0.3 μm. The samples were cleaned with isopropyl alcohol and air dried. Once prepared and free of moisture, the samples were covered with cotton.

The corrosion testing was performed in 250 mL of aqueous electrolyte solution, prepared with 8.75 g of NaCl and 241.25 g of H_2_O, i.e., an equivalent concentration of 3.5 wt% of NaCl [36]. A three-electrode electrochemical cell was used, utilizing a Saturated Calomel Electrode (SCE) as the reference electrode; the auxiliary electrode was a Platinum electrode, and the working electrode was a section of the sample cut from the weld joint.

To evaluate the corrosion results, potentiodynamic curves were analyzed using the Coreware and CorewareView software, while the impedance testing was performed by the ZView and ZPlot software. The test conditions for evaluation through the software were: alloy equivalent weight (9.01), alloy density (2.7 g /cm^2^), Stern–Geary constant (26 mV), and the area of the working electrode, which was estimated from each sample by optical microscope and image analyzer software.

Before starting the test, it is necessary to properly handle the electrochemical cell so that before placing the electrodes, it is required to clean them with distilled water to avoid contamination of the solution. This way, less noise is induced in obtained signals, which are in the order of the millivolt and microampere. On the other hand, once the test has been started, the cell must be undisturbed, and it must be isolated from any vibration [23].

First, for the open circuit test, the potential of the samples was allowed to stabilize for about 15 min in order to have a stable potential (in terms of thermodynamics). Once the polarization potential was stabilized, it was plotted as a function of time for 35 min. Once this lapse was over, the data were processed, obtaining the corresponding graphs, and determining the average open electrical circuit potential.

Then, the potentiodynamic tests were realized. The range analysis for the potentiodynamic tests was from an initial potential of −0.25 V to a final potential of 0.7 V, in reference to the open circuit voltage. The suggested potential sweep is only applicable for AA6061 aluminum alloys, as it is used in Ref. [23], where samples of this same type of alloy were tested. Therefore, in this research, these conditions are also helpful. The scanning speed was set to 1.66 mV/s.

Once the readings from the voltage-current acquisitions were plotted, the linear portion of the anodic and cathodic branches was identified, and straight lines were drawn through the linear parts until they intersected. The intersection coordinates correspond to the corrosion potential (Ecorr, in mV) and the current density (Icorr, in μA/cm^2^). By the ASTM G-102 [36] standard, upon the estimation of the current density, the corrosion rate (CR) in millimeters per year (mmPY), could be estimated as follows (Equation (3)) [23]:

(3) $$CR=K_{1}\left(\frac{Icorr}{\rho }\right)EW$$

where $K_{1}=3.273\times 10^{-3}$ mm·g/μ·A·cm·yr, ρ is the density of the alloy (g/cm^3^), and EW is the equivalent weight (dimensionless).

Finally, to carry out the impedance test in the HAZ, BM, and WZ, the following parameters were used: an initial frequency of 1,000,000 Hz, a final frequency of 1 Hz, and an amplitude of 10 V; then, Nyquist graphs and Bode plots were analyzed [23,36].

## 3. Results

This section presents the results from the performed experimentation: metallographic evaluation, mechanical testing, XRD analysis, and corrosion studies are explained here.

### 3.1. Microstructural Evaluation of the Alloy after the Solubilization Heat Treatment

**Figure 2:**
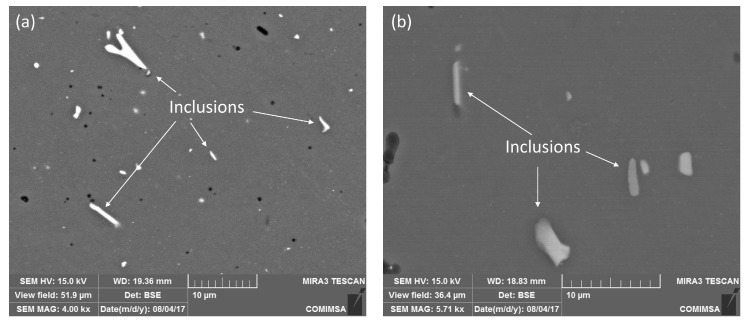
Microstructure of (**a**) AA6061–T6 (as-received) alloy, and (**b**) AA6061–T4 heat-treated alloy.

From metallographic observation, the metallic inclusions were measured on the surface of each alloy: the original AA6061–T6 and the heat-treated AA6061–T4. They exhibited an average size of 3.85 μm for the as-received material (standard deviation, SD = 2.52 μm). In comparison, an average size of 4.12 μm was reported for the heat-treated sample (SD = 2.02 μm). For the original alloy, the volume fraction of the aluminum matrix was measured at 97.64% and 2.35% of metallic inclusions. In contrast, the heat-treated alloy reported 97.03% of aluminum matrix and 2.97% of inclusions. This change of 0.62% in the volume fraction is due to the modification in the distribution of the metallic inclusions in clusters within the aluminum alloy matrix, and the increase in size, after the solubilization heat treatment. This can be observed in Figure 2a,b, for the original alloy and heat-treated sample, respectively, where inclusions have plates, needle, and rod morphologies.

**Figure 3:**
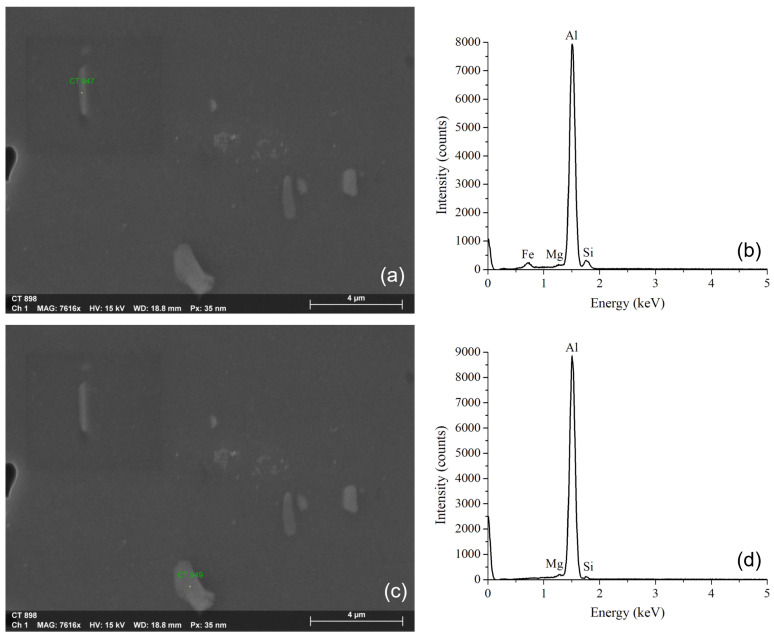
Rod-shaped metallic inclusions (**a**) containing Al, Mg, Fe, and Si, determined by EDS (**b**). The plate-shaped inclusions (**c**) exhibit Al, Mg, and Si in the EDS spectrum (**d**).

Figure 3 shows the results of the EDS analysis performed on specific areas of the metallic particles or inclusions. The presence of Al, Mg, Fe, and Si (Figure 3b) is notable in rod-shaped particles (Figure 3a), as has been reported in [37] using this same technique. Other experts generally identified these particles as Al-Si-Fe intermetallic inclusions in 6061 aluminum alloys [38,39,40]. On the other hand, the intermetallic phase Al-Mg-Si (Figure 3d) has been identified as β (Mg_2_Si) [38,41] in plate-like inclusions (Figure 3c), which corresponds to a hardening phase of the alloy [42].

**Figure 4:**
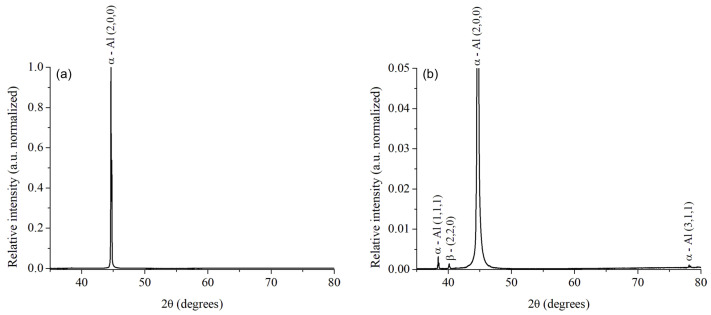
XRD spectra for AA6061–T6 (as-received) alloy at full-scale (normalized) (**a**), and the amplification of the trace peaks (**b**).

**Figure 5:**
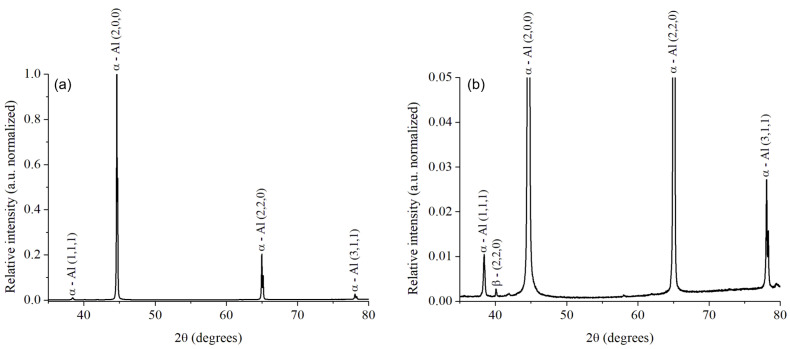
XRD spectra for AA6061–T4 (heat-treated) alloy at full-scale (normalized) (**a**), and the amplification of the trace peaks (**b**).

The diffractogram in Figure 4a exhibited the presence of aluminum for the original AA6061–T6 alloy as a single peak in the (2,0,0) orientation. In the magnification of the spectrum intensity presented in Figure 4b, traces of other peaks of the aluminum matrix in the (1,1,1) and (3,1,1) orientations, and the peak of the β phase Mg_2_Si (2,2,0) were identified. In contrast, in the heat-treated AA6061–T4 alloy, the recrystallization of the aluminum matrix is confirmed by the new aluminum diffraction peak in the (2,0,0) orientation in Figure 5a, and the change in the relative intensity in the other peaks in Figure 5b. The β phase Mg_2_Si (2,2,0) is still present in this alloy. Based on the literature, the β phase is also identified as plate-shaped precipitates [41], as it was exhibited in Figure 3.

### 3.2. Mechanical Testing

**Figure 6:**
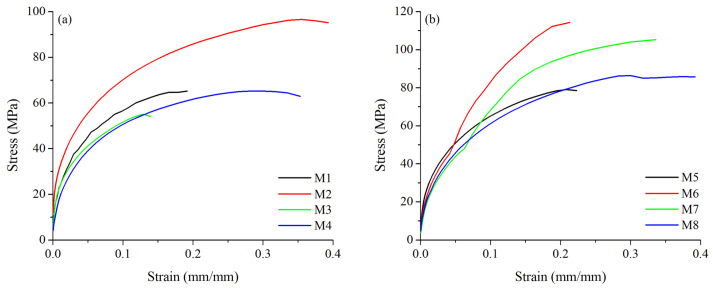
Tensile test curves for AA6061–T6 alloy (**a**), and AA6061–T4 heat-treated alloy (**b**).

The results of the tensile tests are summarized in Table 3; the average of the measurements of each sample and its respective replica is presented. The best results in tensile strength were obtained for sample M6 with heat treatment T4, which reached values of 117 MPa, while for sample M1, in original conditions T6 (as-received, welded with the same processing parameters as M6), a maximum average value of 73.57 MPa was achieved. The worst tensile strength performance was identified for the sample M3, from the original alloy, exhibiting only 54 MPa. Then, it suggests that the heat treatment, together with a good selection of parameters, enhances the weld joint strength. Graphs in Figure 6 show the tensile test curves for AA6061–T6 alloy (a) and AA6061–T4 (b).

Four samples from the tensile strength results were selected to further analysis: the best and the worst sample from each alloy. Consequently, the sample M2 attained the best results from the original AA6061–T6 alloy, reaching 104 MPa, while the worst piece was the M3 from the same alloy with only 54 MPa (overall worst). In the same way, the best sample from the heat-treated alloy AA6061–T4 was sample M6, which reached 117 MPa (overall best), while the worst piece from this heat-treated alloy was sample M8 which exhibited 80 MPa.

**Figure 7:**
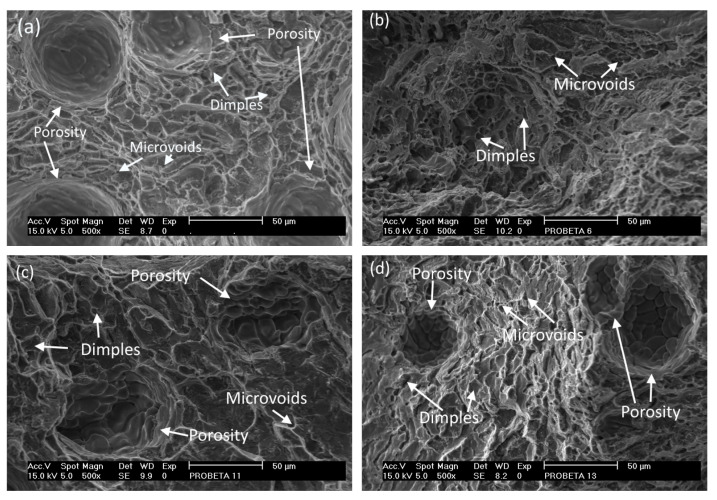
Micrographs in the fracture zone of selected samples of weld joints from AA6061–T6 alloy: (**a**) M2, and (**b**) M3; and weld joints from heat-treated alloy AA6061–T4: (**c**) M6, and (**d**) M8.

The fracture analysis was carried out for the selected samples M2, M3, M6, and M8. The fracture surface in all cases identified a ductile fracture type, which is associated with a mechanism of nucleation and growth of microcavities and, finally, the coalescence of trans-granular micro-dimples [43]. The details can be observed in Figure 7, where microvoids and microcavities are exhibited, which can be classified into fine, medium, and thick [43]; also, dimples and porosity are observed on the fracture surface.

In the GMAW process, the presence of pores is attributed to the entrance of air and moisture since absolute protection of the weld metal from the outside environment is impossible [44]. The pore formation mechanism is attributed to the presence of hydrogen and oxygen in the environment, where factors, such as high temperature and humidity [45], cause some water and oxygen molecules to react with the molten metal and release hydrogen atoms. Hydrogen atoms are easily dissolved in the weld molten at high temperatures; the amount of hydrogen atoms decreases as the temperature decreases, so there is a tendency to have more pores the more input heat is supplied. The solubility of hydrogen in the weld can affect the formation of porosity [46] since the dissolved atoms form bubbles in the weld, and the movement of these atoms is influenced by the flow behavior of the liquid metal. The cooling process reduces the solubility of hydrogen so that the bubbles cannot escape from the weld, thus causing porosity [47,48].

### 3.3. Macrostructural Study and Phase Composition of the Weld Junction

**Figure 8:**
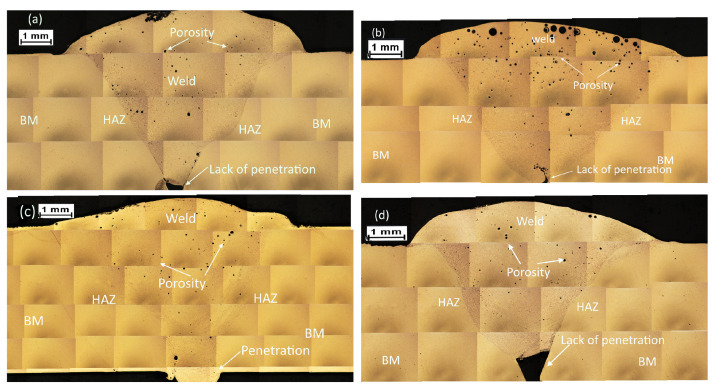
Macrostructure of selected samples: (**a**) M2, the best from AA6061–T6 reached 104 MPa; (**b**) M3, the worst from AA6061–T6, 54 MPa; (**c**) M6, the best from AA6061–T4, 117 MPa; and (**d**) M8, the worst from AA6061–T4, 80 MPa.

For the macrostructural analysis of the weld junctions, micrograph mapping of macro attacks was performed. Based on the tensile strength results, the best and worst conditions of the samples welded by GMAW-P were selected. They are shown in Figure 8, where different zones are identified: BM, HAZ, and WZ, as well as typical weld defects, such as lack of penetration, incomplete fusion, or porosity [29]. In Figure 8a, the sample M2 shows defects, such as incomplete fusion and porosity; sample M3 in Figure 8b exhibits a lack of weld penetration and porosity; sample M6 in Figure 8c, which was the sample with the best mechanical properties, does not present a lack of weld penetration or incomplete fusion, but porosity is observed; finally, the sample M8 in Figure 8d shows incomplete fusion and lack of weld penetration.

The main indicators of the weld quality in the macroscale are the weld crown size, weld penetration, and porosity, as shown in Table 4. From a general point of view, samples of AA6061–T6 exhibited less porosity than samples of AA6061–T4, and the samples with the lowest porosity are M3 for the AA6061–T6 alloy (0.0011 pores/mm^2^) and M5 for the AA6061–T4 (0.0017 pores/mm^2^). However, the samples with the highest tensile strength were M2 and M6, which reported 0.0044 pores/mm^2^ and 0.0065 pores/mm^2^, respectively. Thus, the porosity is not as high to significantly affect the strength of the weld compared to the specimens with the lowest porosity. All weld samples showed adequate weld crown size, considering that the minimum specification is 0.25 mm, and the weld penetration is enough to cover the complete clearance of the joint (plate thickness of 6.4 mm) [29], except for the samples M3 and M8 which presented lack of weld penetration [49].

**Figure 9:**
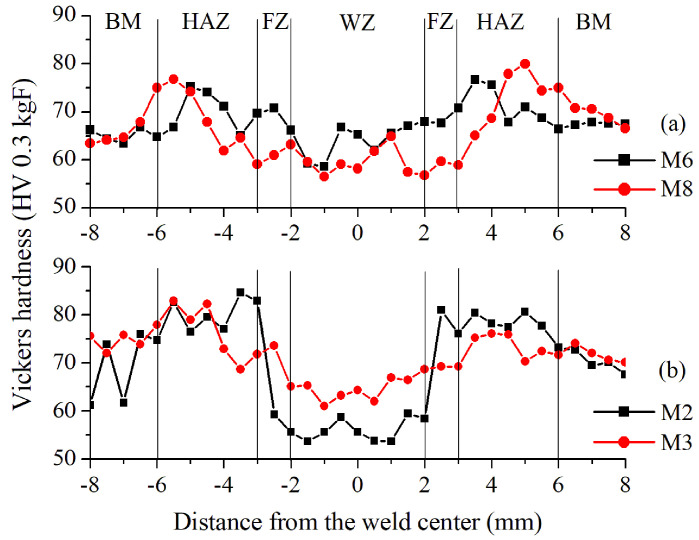
Microhardness profiles for selected samples based on tensile strength performance: (**a**) M6, the best sample, and M8, the worst sample from AA6061–T4 alloy and (**b**) M2, the best sample, and M3 the worst sample from AA6061–T6 alloy.

Microhardness analysis was performed to evaluate its behavior in different zones of the weld joint. First, it is essential to note that the hardness of the base metal for the original alloy AA6061–T6 was measured at an average of 70 HV (SD = 7.5); in contrast, the heat-treated AA6061–T4 material had 65 HV (SD = 5.1), i.e., the solubilization heat treatment reduces the hardness due to the recrystallization of the aluminum matrix. Then, the results of the Vickers hardness measurements in the weld joint zones for the two tested alloys are exhibited in Figure 9.

The AA6061–T4 alloy in Figure 9a presents smother variations in hardness along the weld zones than the AA6061–T6 sample in Figure 9b. The variation range for the pieces in the original T6 condition is between 54 and 85 HV for the sample M2 and 61 to 83 HV for M3. On the other hand, the samples in the T4 heat treatment condition present a hardness between 59 to 77 HV for sample M6 and 57 to 80 HV for sample M8. In both conditions, T6 and T4, the WZ exhibits a trend to present the lowest hardness values, but the samples in the original material give the lowest values in this zone (54 HV for sample M2). Contrarily, the HAZ exhibits the highest hardness values, as previously reported in the literature [50], reaching the highest hardness for the original T6 alloy with up to 85 HV in sample M2.

The low hardness values in the WZ are attributed to the filler metal used in the welding process. However, to understand the increase in the hardness of the HAZ, a detailed diffraction study was conducted for this zone. The Rietveld technique was utilized to quantify the constituent crystalline phases in the HAZ on the near-surface region. This method, known as a technique for refinement of a crystalline structure, consists of a theoretical adjustment of the diffraction patterns, applying a model that includes structural and experimental factors; the reference parameters given at the beginning of the test are modified by adjusting the complete profile of the diffraction pattern of the sample [34].

**Figure 10:**
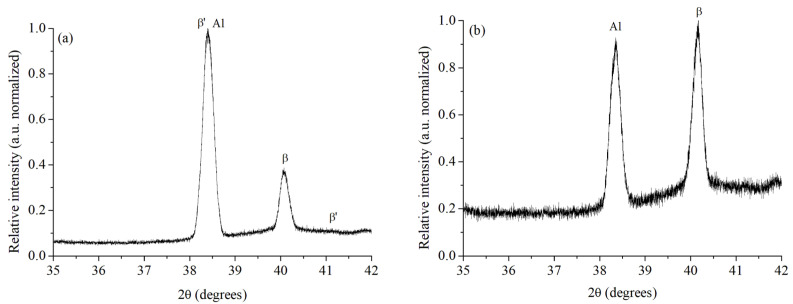
Microdiffraction patterns in the HAZ for selected samples (**a**) M2 in the original AA6061–T6 condition, and (**b**) M6 heat treated AA6061–T4. The peak intensity is normalized to the most relevant peak.

The best-evaluated samples from the tensile tests, M2 (AA6061–T6) and M6 (AA6061–T4) were selected for analysis with XRD in microdiffraction mode. The microdiffraction spectra are exhibited in Figure 10. For the sample M2, the β phase (Mg_2_Si) was detected in a phase percentage of 25.6%, β’ (Mg_9_Si_5_) represented 2.1%, while the remaining 72.4% corresponds to Al. The microdiffraction pattern of this sample is exhibited in Figure 10a. In contrast, in the sample M6, 48% of β and 52% of Al were estimated for the microdiffraction pattern shown in Figure 10b.

The results of the Rietveld refinement are shown in Table 5 for the original AA6061–T6 alloy and in Table 6 for the heat-treated AA6061–T4 alloy. The β phase (Mg_2_Si) exhibits a cubic crystal system in the Fm-3m space group (ICDD chart Ref. Code 01-014-4260) [51]. The phase β’ (Mg_9_Si_5_) presents a hexagonal structure in the P62/m space group (ICDD chart Ref. Code 04-020-3391) [52]. Finally, the aluminum matrix has a cubic crystal system (ICDD chart Ref. Code 01-089-2837) [53].

The Rietveld refinement technique allows the approximate quantification of the different phases on the near-surface of the HAZ, although some of them could be present in the same orientation of the matrix and their lattice d-spacing is similar to the aluminum crystal structure (see Table 5 and Table 6). In the literature, the precise observation of certain phases was performed only with Transmission Electron Spectroscopy [54]. However, the hardness behavior also suggests the presence of these phases and, together with XRD results, the dissolution process of the precipitates explains the diminishing hardness observed in the WZ (Figure 9) due to the reduction in obstacles to the dislocation movement promoted by the weld heating [55]. Meanwhile, the reprecipitation of phases driven by Mg/Si-rich particles occurs in the vicinity of the HAZ [54]. In this region, the relatively high hardness is attributed to the GP zones, which are coherent with the Al matrix and have similar lattice parameters [56].

**Figure 11:**
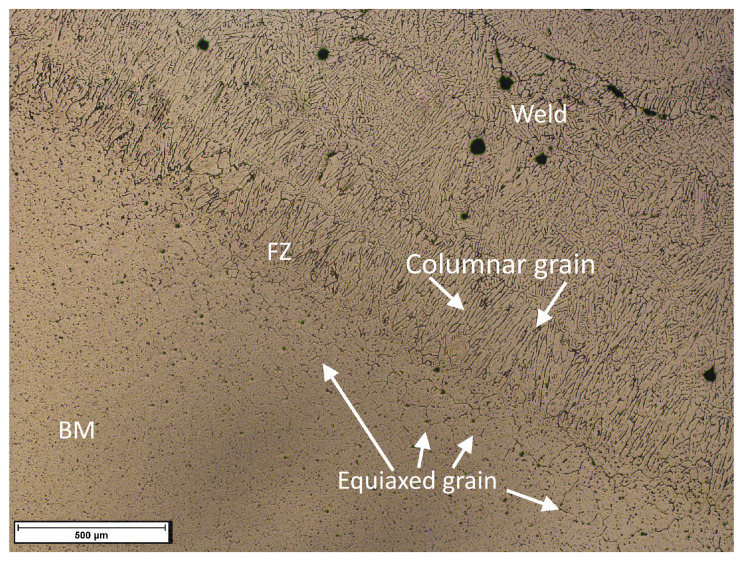
Heterogeneous grains in different zones of the weld joint.

The grain structure in the different joint zones also plays an important role in hardness (see Figure 11). The base metal contains coarse, elongated equiaxed grains with uniformly distributed precipitates. The weld shows dendritic structures formed during the heating of the base metal due to heat from the welding process and the rapid cooling of the molten metal [57]. Elongated equiaxed grains can be observed in the fusion zone, which influences the hardness and tensile strength of the joint without neglecting the presence of porosity. As can be seen in the micrograph, the disparity of equiaxed and columnar grains influences the changes in microhardness, in addition to the fact that a typical dendritic structure can be seen in the welding area. These changes are attributed to the heat distribution in the sample’s zones during the welding process and the different cooling velocities [22,58,59].

### 3.4. Corrosion Tests

The corrosion tests were carried out for the selected samples M2, M3, M6, and M8: the best and worst conditions based on the tensile strength results, as mentioned before. Results of the open-circuit polarization, potentiodynamic polarization, and electrochemical impedance spectroscopy are summarized in Table 7. All corrosion tests were performed in the different zones of the weld joint: BM, HAZ, and WZ. Sections from the different weld zones were cut using a precision diamond disk to extract a representative portion for the corrosion study. The area of these sections is reported in the last column of Table 7, and it is required to estimate the corrosion parameters.

The macro-scale analysis of the welding quality parameters (see Table 4) reveals that the M3 sample presented the lowest porosity with 0.0011 pores/mm${}^{2}$. This sample shows the best values of all the samples evaluated in corrosion rate for all zones of the joint: 0.017 mmPY in the base metal, 0.012 mmPY in the HAZ, and 0.020 mmPY in the welding zone. In contrast, sample M6 reported the highest porosity of all samples with 0.0068 pores/mm${}^{2}$, and the reported corrosion rate was 0.027 mmPY in the base metal, 0.050 mmPY in the HAZ, and 0.034 mmPY in the weld zone. Then, the porosity seems to increase the corrosion susceptibility.

It is known that the presence of porosity in the weld area can cause a decrease in mechanical properties, such as stress and fatigue, and can also potentially affect corrosion performance [60]. The leading cause of corrosion in this type of welding is the galvanic couple that forms between the intermetallic base Fe and the Mg_2_Si phases, and we associate the pores because they cause a concentration of stress and the loss of coherence of the matrix during and after the process of solidification and pore formation [46].

**Figure 12:**
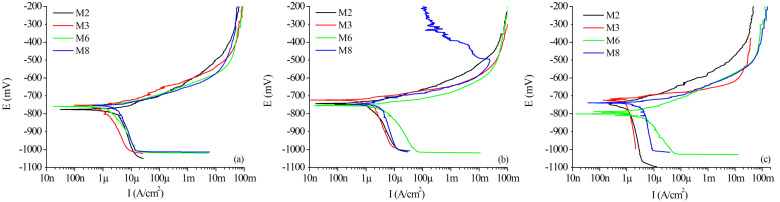
Evans diagrams for selected samples in different zones: (**a**) Base Metal, (**b**) Weld Zone, and (**c**) Heat-Affected Zone.

Figure 12a compares the Evans diagram exhibiting the relationship of current and potential for oxidation and reduction reaction from the potentiodynamic polarization test for the base metal of selected samples. The corrosion potential (Ecorr) for the base metal of all samples was relatively close to each other. The values of Ecorr for M2, M3, M6, and M8 were −776, −770, −762, and −767 mV, respectively (see Table 7). On the other hand, after applying the Tafel technique, the current density (Icorr) was estimated in 3.41, 1.58, 2.48, and 3.50 μA/cm^2^, in the same order of samples. This resulted in a different corrosion rate (CR, Equation (3)) for the base metal of the samples, whose values were 0.037, 0.017, 0.027, and 0.038 mmPY, respectively. Although the base metal for sample M2 is the same as sample M3, and, similarly, M6 and M8 have the same alloy, the corrosion rate is different because the sectioning of the base metal was performed close to the weld joint; then, the heat from the process could affect the corrosion resistance near to this region. In this sense, sample M2, which received more heat (1297 J/mm, from Table 7) than sample M3 (1169 J/mm), presented more corrosion susceptibility. Additionally, sample M8 received more heat from the process (1176 J/mm) than sample M6 (1097 J/mm); then, the corrosion performance on the base metal of M8 was better than M6.

From the Evans diagrams in Figure 12b, recorded in the welding area, it is observed that the highest potential (the more positive) is attributed to the sample M3, with Ecorr = −723 mV, and the current density Icorr = 1.79 µA/cm${}^{2}$. However, the corrosion rate for the welding zone of the samples from the original alloy (M2 and M3) is very similar, resulting in values of 0.018 and 0.020 mmPY, respectively. In contrast, the samples from the heat-treated alloy (M6 and M8) reported 0.034 and 0.041 mmPY, respectively, twice the corrosion rate obtained in the original alloy.

Finally, the Evans diagrams in Figure 12c exhibit the behavior in the HAZ for the selected samples. Those from the original alloy reported the overall best results, with 0.012 and 0.014 mmPY for samples M3 and M2, respectively. On the opposite, the heat-treated alloy reported the overall worse results, with 0.048 and 0.050 mmPY for samples M8 and M6, respectively.

Fahimpour et al. [23] obtained similar results, determining that materials that are subjected to a welding process, due to the application of heat, suffer lower corrosion resistances, obtaining similar trends to those reported here in the values of Ecorr and Icorr after TIG and FSW processes.

Electrochemical impedance spectroscopy is a technique that provides complementary information about the corrosion phenomena of the analyzed materials. Evaluating the results of polarization resistance (Rp) for the base metal under the different welding and heat treatment parameters, a trend to increase Rp at higher heat inputs is noted for both alloys (see Table 7). The base metal of the sample M3, welded with 1169 J/mm, obtained 12.56 kΩ, and the sample M2, welded with 1297 J/mm, reached 16.89 kΩ. Similarly, sample M6 (1097 J/mm) resulted in 17.57 kΩ, and sample M8 (1176 J/mm) resulted in 28.78 kΩ.

However, the opposite behavior occurred in the WZ and HAZ of all samples, i.e., as the higher the heat input, the lower the value of Rp. The WZ of sample M3 (1169 J/mm) recorded 44.49 kΩ, but the sample M2 (1297 J/mm) obtained 29.43 kΩ. This behavior was also observed in the WZ of the AA6061–T4 alloy, where the sample M6 (1097 J/mm) registered 239.17 kΩ, almost twice the value of sample M8 (1176 J/mm), which obtained 129.60 kΩ. Comparably, the HAZ of all samples recorded a reduction in the Rp value when the heat input increased. The sample M3 (1169 J/mm) resulted in 90.71 kΩ, but sample M2 (1297 J/mm) reached 2.15 kΩ only. Additionally, sample M6 (1097 J/mm) achieved 54.50 kΩ, and 48.33 kΩ were obtained for sample M8 (1176 J/mm).

The behavior of reduction in the Rp due to an increase in the heat input could be attributed to the fact that the heat input forces the alloying elements to form harder precipitates, as was commented during the microstructural evaluation of the samples. It can help to increase the mechanical resistance, but in terms of corrosion, this generates thermodynamically unstable zones favoring the corrosion phenomenon [61,62]. WZ and HAZ become the most critical parts of the weld joint since they broadly define the mechanical resistance of the component but are the primary receptors of the effects of the heat.

The solution resistance (Rs), i.e., the equivalent resistance of the electrolyte, remained stable and between the expected values for all experiments. This is because the proportion of NaCl was not changed during the tests. For some cases, the value of Rs was higher due to the presence of oxides from the sample’s dissolution.

**Figure 13:**
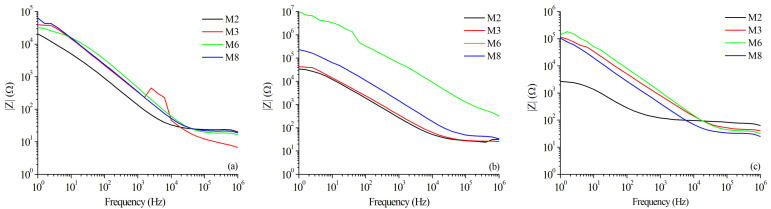
Magnitude in Bode plots for selected samples in different zones: (**a**) Base Metal, (**b**) Weld Zone, and (**c**) Heat-Affected Zone.

The results of the equivalent Rp were confirmed by the magnitude of impedance versus frequency in the Bode plots exhibited in Figure 13a, for the BM zone, Figure 13b for the WZ, and Figure 13c for the HAZ. The trend to a lower resistance at higher heat inputs for the WZ (Figure 13b) and HAZ (Figure 13c) is also noted in these plots. The high impedance values at high frequencies are attributed to the passive oxide layer, which is considered a parallel circuit formed by a resistor (due to ionic conduction in this layer) and a capacitor (due to its dielectric properties). Moreover, because of the heterogeneity of the phases in the HAZ, it could be more susceptible to corrosion due to the residual stresses in this area [63,64].

**Figure 14:**
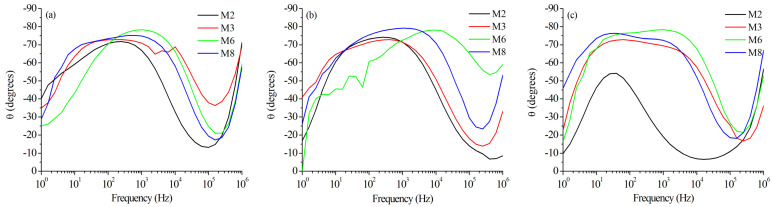
Phase in Bode plots for selected samples in different zones: (**a**) Base Metal, (**b**) Weld Zone, and (**c**) Heat-Affected Zone.

The magnitude of impedance presents two different regions, presumably because there are two separate corrosion mechanisms: the first is associated with uniform corrosion, and the second could represent localized corrosion. The capacitive effect of the double-layer cell is notorious because of the low impedance at high frequencies, followed by the high impedance at low frequencies (near the limit of the detectability of the test). The phase versus frequency in the Bode plots of Figure 14 confirms the described effect of two corrosion mechanisms, where the phase angle starts with low values at low frequencies, stabilizing its behavior in the mid-range of frequencies (predominant capacitive effect), then reaching low values and exhibiting a new increase in the angle at the higher values of frequency. The same behavior was observed in the three studied zones (BM, WZ, and HAZ) of all samples.

**Figure 15:**
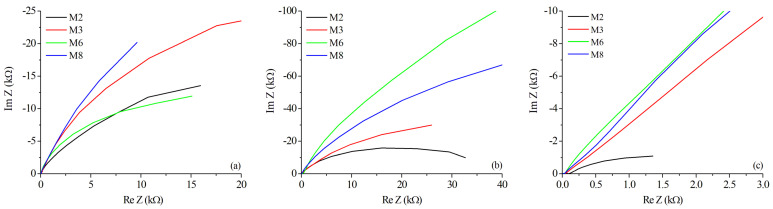
Nyquist diagrams for selected samples in different zones: (**a**) Base Metal, (**b**) Weld Zone, and (**c**) Heat-Affected Zone.

The Bode plots of the magnitude of impedance and phase as a function of frequency are in accordance with those reported in the literature [63]. However, the Bode plots do not allow establishing the electrochemical behavior of the tested samples without the additional information obtained from the Nyquist diagrams, exhibited in Figure 15 for the selected samples M2, M3, M6, and M8, in the three essential weld regions, BM, WZ, and HAZ.

The real versus imaginary impedance values attained in the HAZ, compared to the WZ and BM, provide information to determine that the HAZ is the most susceptible region to electrochemical corrosion. Nyquist plots of Figure 15c showed that the HAZ reached the lowest impedance in all samples compared to the other zones. Moreover, except for sample M2, the rest of the pieces appear to have the typical form of a Warburg impedance, i.e., the curves are close to linear, reflecting the strong ionic transference from the tested piece to the working electrode, in almost a constant rate, almost independently of the frequency [61,62,64,65,66]. The BM presents the best corrosion performance of all zones, exhibiting a trend to form semicircular loops, as observed in Figure 15a, mainly in sample M3, which reached the lowest corrosion rate of BM samples during the polarization tests. Finally, the WZ in Figure 15b exhibited a semicircular graph for samples M2 and M3, demonstrating a better corrosion performance than M6 and M8, which reported a more significant imaginary component in the impedance; also, this agrees with the lower corrosion rates reported for samples M2 and M3, in comparison to samples M6 and M8 in the welding zone.

## 4. Discussion

The mechanical strength and corrosion performance in the weld joint of AA6061 aluminum alloy by pulsed gas metal arc welding were evaluated in this research. This study comprised two variations in the heat treatment of the alloy; the first, in the original condition (as received), defined as T6, and the second after a solubilization heat treatment designated as T4, which potentially improves the mechanical properties and weldability of the alloy due to the redistribution of the hardening phases within the aluminum matrix.

After the welding process, the tensile strength of the weld joint reached 117 MPa in the heat-treated alloy (AA6061–T4) when a moderate heat input was used, i.e., an adequate combination of polarization voltage, weld current, and welding speed, overcoming the best result in the original alloy (AA606–T6), which recorded a maximum of 104 MPa. Chikhale et al. [20], and Kaushal et al. [67] obtained tensile strength values from 84 to 136 MPa, applying 160 A, determining that this moderate amperage and a slow welding application speed produce a coarsening of the grains in the welding zone of the samples, thus improving the mechanical resistance of the weld. Consequently, a good combination of welding parameters and heat treatment results in the strengthening of the weld joint.

However, more than an overall evaluation of the weld joint, it is convenient to analyze the performance of each welding region involved in the structure: the base metal, the heat affected zone, and the welding zone, for a clearer understanding of the phenomena associated with each region. As mentioned before, the condition of the base metal has an influence on the weldability and strength of the weld joint because of the formation of precipitates of the β hardening phase [5,68], and the recrystallization of the aluminum matrix, as the XRD analysis and the microstructural evaluation of the utilized alloys demonstrated it. However, the other zones are equally important.

In our observations, the fracture zone in all samples evaluated in the tensile test was near the fusion zone, i.e., the interface region between the base metal and the weld zone. The heat transferred from the welding machine melts the filler metal to form the welding zone, but much of this heat is transferred to the base metal also, thus producing a heat-affected area (HAZ). Dawood et al. [50] and Sevim et al. [49] identified the cause of the failure nearby the fusion zone to the growth of elongated equiaxed grains attributed to the high temperature from the process, causing the dissolution of precipitates, thus forming zones of low and high hardness. The latter was also detected in our tests, where the HAZ presented high hardness, followed by low hardness in the welding zone, being more notorious in the original alloy than in the heat-treated one.

The redistribution of precipitates in the HAZ formed a high content of the β phase in this zone of the heat-treated alloy, while β and β’ was observed in the HAZ of the as-received material, as was determined by the microdiffraction analysis and the use of Rietveld refinement technique. Following Sevim et al. [49], the heat from the process causes the recrystallization of the grains in the HAZ, which increases the hardness in this zone, while a lower hardness occurs in the fusion zone, which provides ductility properties due to the increased grain size. These transitions in the microstructure manifested as hardness changes, as well as the changes in the phase composition, cause the ductile failure observed in all samples of our study [43].

According to the literature, the changes in the microstructure of the base metal, heat-affected zone, and welding zone cause a detriment in the corrosion resistance due to the heterogeneous and multiphase structures [66,69]. The potentials of the distinct phases are different, making a natural galvanic couple susceptible to electrochemical corrosion effects. Si-rich and Cu-rich phases are the best examples [23,64]; the potential difference with respect to the aluminum matrix causes corrosion cells. High amounts of these precipitates (different forms of the β phase) cause relevant cathodic reactions. For this reason, we analyze the corrosion performance of the different zones of the weld joint.

Zones with differences in grain size are also more susceptible to electrochemical corrosion. This is represented in the heat-affected zone; as it has been shown in this zone, there is a difference in grain size between the base metal, the heat-affected area, and the weld. In addition, the heat-affected zone is the region with the highest stress concentrations [66,70]. Consequently, this zone resulted in the area with the highest corrosion rates and the greater susceptibility to present Warburg impedance, mainly in the heat-treated alloy.

In the literature, it has been shown that small grain sizes increase the number of grain boundaries, which are thermodynamically susceptible to corrosion and cause poor corrosion resistance behavior [23]. The formation of small grains in the heat-affected zone (see Figure 11) due to recrystallization caused by the welding process increases the number of grain boundaries, generating greater stress concentration and accumulation of dislocations compared to the coarse grains of the base metal [71]. Moreover, during thermal cycles, Fe particles dissolve at the grain boundaries causing a lower resistance to corrosion in small grains regions than in coarse grain zones [23,71].

Zhou et al. [39] described the corrosion mechanism as the interaction of Mg_2_Si particles with Al-Fe-Si intermetallics, which cause micro galvanic corrosion due to the heterogeneity of microstructures and phases. Gharavi et al. [61] defined the Mg_2_Si phase as the cathode and the Fe-rich intermetallics as the anode. Large amounts of these precipitates cause more cathodic reactions [23], which cause electrochemical corrosion cells due to the potential difference between the aluminum matrix and the precipitates rich in Fe and Mg_2_Si phases. The heat generated during the process causes a dissolution of the Mg_2_Si phase and the multiphase Fe particles, promoting localized corrosion, accentuated by the existence of pores, as our results have suggested, which leads to the generation of pitting corrosion. There is also documented evidence to determine that micro galvanic corrosion predominates in the welding zone due to the distribution of intermetallic Fe in this zone [38].

Finally, the effect of the heat input on the behavior of the corrosion performance of the distinct zones of the weld joint is remarkable. Mainly, the development of the electrochemical corrosion in the welding zone and heat-affected zone is notorious, where the increase in the heat causes a decrease in the corrosion performance, associated with the formation of thermodynamically unstable zones [61,62], where both uniform and localized corrosion occur.

## 5. Conclusions

In this paper, we present the results and analysis of the evaluation of electrochemical corrosion and mechanical testing of a weld joint in aluminum type 6061 alloy. The joint was produced by gas metal arc welding in pulsed metal transfer mode. The main conclusion of the research was that the heat input influences the mechanical strength of the joint, making it stronger with a good selection of processing parameters but negatively affecting its corrosion performance, mainly in the heat-affected zone. This is attributed to the formation of galvanic couples in the weld due to variations in precipitate distribution. Additionally, the appearance of zones of high and low hardness due to the heat of the process promotes the fracture of the specimens near the fusion zone.

The heat input is an essential factor in the polarization resistance response in electrochemical corrosion since samples with lower heat input had higher mechanical strength but better polarization resistance. The heat-affected zone is the area with the most significant susceptibility to corrosion due to the stresses concentrated in that area and its difference in hardness compared with the weld zone and the base metal.

The solubilization heat treatment favors a better mechanical strength, but it has controversial effects on the corrosion resistance of the weld joint. In the polarization resistance, the heat treatment benefits the increase in this value, suggesting that the formation of the galvanic couples is less significant than in the samples of the original material. However, evaluating the corrosion rate, the original alloy generally presents the best performance. This is attributed to the mixed mechanisms of uniform and localized corrosion; the latter is favored by pores and defects.

Further investigations should be conducted to evaluate a better control of the heat input because this is a crucial parameter to obtaining high-quality weld joints. The latter implies the simultaneous manipulation of voltage, current, and welding speed, but this is a condition that commercial equipment does not usually offer. Moreover, different filler metals and other pre- or post-heat treatments could be studied. Finally, the implications of the corrosion performance and the use of other solutions or electrolytes must be addressed to focus on the potential applications of this alloy.

## Figures and Tables

**Table 1:** Chemical composition of the AA6061 alloy and the filler compound 4043–ER.

Alloy	UNS No.	ISO No.	Composition wt. % (Al, Balance)									
			Si	Fe	Cu	Mn	Mg	Cr	Zn	Ti	Others	
											Indiv.	Total
4043–ER	A94043	AlSi_5_	6.00	0.80	0.30	0.05	0.05		0.10	0.20	0.05	0.15
AA6061–T6	A06061	AlMgSiCu	0.80	0.70	0.40	0.15	1.20	0.35	0.25	0.15	0.05	0.15

**Table 2:** Experimental parameters for the GMAW-P welding process.

ID	HT	*I* (Amperes)	*V* (Volts)	WS (mm/min)	Qnet (J/mm)
M1	T6	250	25	318	1014
M2	T6	175	25	174	1297
M3	T6	212	25	234	1169
M4	T6	280	25	366	987
M5	T4	212	25	216	1266
M6	T4	250	25	294	1097
M7	T4	280	25	336	1075
M8	T4	175	25	192	1176

**Table 3:** Mechanical properties from the tensile test of welded samples. SD in parenthesis.

	Yield	Ultimate	Rupture	Young	
	Strength	Strength	Load	Modulus	Elongation
ID	(MPa)	(MPa)	(N)	(MPa)	(%)
M1	22 (2.4)	74 (11.8)	5449 (544)	2925 (139)	14 (9.9)
M2	40 (3.2)	104 (11.0)	7139 (211)	1146 (3.53)	40 (1.4)
M3	30 (4.4)	54 (2.1)	4048 (2.12)	1316 (75.7)	15 (0.7)
M4	36 (2.1)	65 (0.6)	4906 (149)	654 (106)	35 (2.1)
M5	33 (1.3)	83 (5.0)	6128 (19.8)	813 (47.3)	25 (1.4)
M6	79 (0.9)	117 (4.5)	8793 (83.4)	568 (82.7)	43 (26.9)
M7	40 (1.1)	105 (0.5)	7802 (240)	850 (35.1)	39 (2.1)
M8	30 (0.8)	80 (3.8)	6577 (40.3)	2972 (242)	24 (24.0)

**Table 4:** Evaluation of weld crown, weld penetration, and porosity in the welded samples.

	Weld Crown	Weld Penetration	Porosity
ID	(mm)	(mm)	(pores/mm^2^)
M1	1.1	6.6	0.0065
M2	1.8	6.7	0.0044
M3	1.4	6.0	0.0011
M4	0.7	6.5	0.0023
M5	2.1	6.6	0.0017
M6	1.3	9.0	0.0068
M7	0.8	8.1	0.0067
M8	1.7	6.1	0.0048

**Table 5:** Summary of the Rietveld refinement of microdiffraction spectrum in the HAZ of the sample M2 (as-received alloy).

Position	Height	FWHM	d-Spacing	Rel. Int.	Orientation	Phase
(° 2θ)	(cps)	(° 2θ)	(Angstrom)	(%)		
36.0746	28.59	0.0864	2.48775	1.80	2 0 3	β’
38.2650	18.72	0.0858	2.35023	0.88	1 2 0	β’
38.4093	2139.19	0.1939	2.34174	100	1 1 1	α-Al
40.0805	781.91	0.1981	2.24786	36.55	2 2 0	β
41.1628	13.10	0.0845	2.15826	0.25	1 2 2	β’

**Table 6:** Summary of the Rietveld refinement of microdiffraction spectrum in the HAZ of the sample M6 (heat-treated alloy).

Position	Height	FWHM	d-Spacing	Rel. Int.	Orientation	Phase
(° 2θ)	(cps)	(° 2θ)	(Angstrom)	(%)		
38.3134	603.63	0.2113	2.34838	100	1 1 1	α-Al
40.1158	597.2	0.2078	2.24597	98.94	2 2 0	β

**Table 7:** Corrosion results for open-circuit potential, potentiodynamic polarization, and electrochemical impedance spectroscopy for selected samples.

	OCP	Ecorr	Icorr	Corr. Rate	R_s_	R_p_	C_dl_	Area
Zone	(mV)	(mV)	(μA/cm^2^)	(mmPY)	(Ω/cm^2^)	(kΩ/cm^2^)	(μF/cm^2^)	(cm^2^)
M2 BM	−790	−776	3.41	0.037	26.33	16.89	1.710	0.47
M2 HAZ	−746	−754	1.28	0.014	95.99	2.15	7.710	0.08
M2 WZ	−765	−741	1.61	0.018	30.18	29.43	0.702	0.13
M3 BM	−766	−770	1.58	0.017	29.63	12.56	0.554	0.13
M3 HAZ	−774	−721	1.09	0.012	49.33	90.71	0.238	0.05
M3 WZ	−746	−723	1.79	0.020	29.38	44.49	0.556	0.12
M6 BM	−765	−762	2.48	0.027	18.88	17.57	0.382	0.35
M6 HAZ	−786	−806	4.59	0.050	40.17	54.50	0.151	0.13
M6 WZ	−767	−750	3.15	0.034	406.90	239.17	0.0018	0.15
M8 BM	−765	−767	3.50	0.038	21.78	28.78	0.552	0.30
M8 HAZ	−763	−764	4.38	0.048	34.45	48.34	0.480	0.14
M8 WZ	−757	−743	3.53	0.041	42.87	129.60	0.122	0.10

## Data Availability

Not applicable.

## Abbreviations

The following abbreviations are used in this manuscript:

BM	Base Metal (also identified as parent metal)
FSW	Friction Stir Welding
FZ	Fusion Zone
GMAW	Gas Metal Arc Welding
GMAW-P	Pulsed Gas Metal Arc Welding
GP	Guinier–Preston Zones
HAZ	Heat-Affected Zone
HV	Hardness Vickers
OCP	Open-Circuit Potential
SD	Standard Deviation
SEM	Scanning Electron Microscope, Scanning Electron Microscopy
TIG	Tungsten Inert Gas
WZ	Welding Zone
XRD	X-ray Diffraction, X-ray Diffractometer
